# Meta-analysis of the normal diffusion tensor imaging values of the peripheral nerves in the upper limb

**DOI:** 10.1038/s41598-023-31307-2

**Published:** 2023-03-24

**Authors:** Ryckie G. Wade, Fangqing Lu, Yohan Poruslrani, Chiraag Karia, Richard G. Feltbower, Sven Plein, Grainne Bourke, Irvin Teh

**Affiliations:** 1 Leeds Institute for Medical Research, The Advanced Imaging Centre, Leeds General Infirmary, University of Leeds, Leeds, LS1 3EX UK; 2 Department of Plastic and Reconstructive Surgery, Leeds Teaching Hospitals Trust, Leeds, UK; 3 Royal College of Surgeons in Ireland, University of Medicine and Health Sciences, Dublin, Ireland; 4 Leeds Institute for Data Analytics, University of Leeds, Leeds, UK; 5 Leeds Institute for Cardiovascular and Metabolic Medicine, University of Leeds, Leeds, UK; 6 The Advanced Imaging Centre, Leeds Teaching Hospitals Trust, Leeds, UK

**Keywords:** Diagnostic markers, Peripheral neuropathies, Peripheral nervous system, Biological physics

## Abstract

Peripheral neuropathy affects 1 in 10 adults over the age of 40 years. Given the absence of a reliable diagnostic test for peripheral neuropathy, there has been a surge of research into diffusion tensor imaging (DTI) because it characterises nerve microstructure and provides reproducible proxy measures of myelination, axon diameter, fibre density and organisation. Before researchers and clinicians can reliably use diffusion tensor imaging to assess the ‘health’ of the major nerves of the upper limb, we must understand the “normal” range of values and how they vary with experimental conditions. We searched PubMed, Embase, medRxiv and bioRxiv for studies which reported the findings of DTI of the upper limb in healthy adults. Four review authors independently triple extracted data. Using the meta suite of Stata 17, we estimated the normal fractional anisotropy (FA) and diffusivity (mean, MD; radial, RD; axial AD) values of the median, radial and ulnar nerve in the arm, elbow and forearm. Using meta-regression, we explored how DTI metrics varied with age and experimental conditions. We included 20 studies reporting data from 391 limbs, belonging to 346 adults (189 males and 154 females, ~ 1.2 M:1F) of mean age 34 years (median 31, range 20–80). In the arm, there was no difference in the FA (pooled mean 0.59 mm^2^/s [95% CI 0.57, 0.62]; I^2^ 98%) or MD (pooled mean 1.13 × 10^–3^ mm^2^/s [95% CI 1.08, 1.18]; I^2^ 99%) of the median, radial and ulnar nerves. Around the elbow, the ulnar nerve had a 12% lower FA than the median and radial nerves (95% CI − 0.25, 0.00) and significantly higher MD, RD and AD. In the forearm, the FA (pooled mean 0.55 [95% CI 0.59, 0.64]; I^2^ 96%) and MD (pooled mean 1.03 × 10^–3^ mm^2^/s [95% CI 0.94, 1.12]; I^2^ 99%) of the three nerves were similar. Multivariable meta regression showed that the b-value, TE, TR, spatial resolution and age of the subject were clinically important moderators of DTI parameters in peripheral nerves. We show that subject age, as well as the b-value, TE, TR and spatial resolution are important moderators of DTI metrics from healthy nerves in the adult upper limb. The normal ranges shown here may inform future clinical and research studies.

## Introduction

Peripheral neuropathy and nerve injury are common, affecting approximately 1 in 10 adults over the age of 40 years^1^. Given the absence of a reliable diagnostic test, there has been a surge of research related to diffusion-weighted magnetic resonance imaging (dMRI) for the evaluation of peripheral nerves. dMRI characterises tissue microstructure and provides reproducible^2–5^ proxy measures of nerve health which are sensitive to axon type, diameter, density, myelination and organisation^6–9^.

The most prevalent form of dMRI in peripheral nerves is diffusion tensor imaging (DTI). This typically generates the following voxel-wise parameters; fractional anisotropy (FA), mean diffusivity (MD), axial diffusivity (AD) and radial diffusivity (RD). FA is a scalar value between zero and one; an FA of zero implies isotropic diffusion within a voxel, whilst (in the context of peripheral nerves) a FA nearing one implies diffusion predominantly along a single axis (i.e., axoplasmic along nerves). MD describes the average molecular diffusion rate of the tensor; AD describes the diffusion rate in the long axis and RD represents diffusion perpendicular to the long axis.

The diffusion of water and therefore dMRI signal is affected by factors other than tissue microstructure and physiology. Studies in the brain have shown that dMRI outputs are dependent on scanner hardware, acquisition settings, preprocessing techniques, reconstruction algorithms and extraction methods^2,10–15^. Recent work has shown similar dependence in the brachial plexus^16^ and median nerve in the hand^17^. Therefore, before researchers and clinicians can reliably use dMRI to assess the ‘health’ of the major nerves of the upper limb, there is a need to define the range of “normal” values and how they vary with experimental conditions. These knowledge gaps form the rationale for this review.

## Methods

This review was registered on the PROSPERO database (CRD42021275343). It was designed and conducted in accordance with the Cochrane Handbook of Systematic Reviews^18^ and reported in accordance with the PRISMA checklist^19^.

### Types of studies

We included studies which reported the findings of DTI of the arm, elbow or forearm in healthy adults. There were no language restrictions. Case reports were excluded.

### Participants

This review considers adults (aged ≥ 16 years) with no known pathology (past or present) affecting any peripheral nerve(s) of the upper limb.

### Image acquisition

The included studies must have reported the DTI parameters from any of the median, ulnar or radial nerve in the forearm. The forearm was defined as the anatomical region distal to the elbow joint and proximal to the radiocarpal joint.

### Search strategy

In accordance with our search strategy (Appendix 1), PubMed and Embase were interrogated using the NICE Healthcare Databases (hdas.nice.org.uk), and medRxiv and bioRxiv were searched using the R package medrxivr^20^ from inception to the 5th January 2022. This yielded 93 hits in PubMed, 509 in Medline but none in the preprint archives. Later, CitationChaser^21^ was used for forward and backward chasing of 452 citations, which yielded a further 371 records on 28th January 2022.

### Study selection

Three review authors (FL, YP and RGW) independently screened titles and abstracts for relevance, in accordance with the eligibility criteria. The full texts of potentially eligible articles were obtained and again independently assessed by the same authors. Disagreements were resolved by discussion.

### Data extraction

Three review authors (FL, YP and RGW) independently extracted data in duplicate, after which a 4th review author (CK) independently performed complete data validation (i.e. triplicate extraction). Where bilateral or repeated (e.g., test–retest) measurements were reported, the values were averaged given that the variability of DTI metrics from peripheral nerves on the right and left side^22–24^, intrasessional and intersessional variability is less than 5%^25^. When data were missing or unclear, the corresponding author was contacted by email and if no reply was received, values were back-calculated^26^ or extracted from graphs using metaDigitise^27^. Some studies used the term apparent diffusion coefficient (ADC) rather than MD. By convention, MD is used to describe the average of the diffusion tensor eigenvalues in DTI. As all the included studies fitted DTI models to their data, we refer to their reported results as MD rather than ADC. Two authors provided additional information upon request^28,29^.

### Outcomes

We planned to estimate the normal FA and diffusivity values (MD, RD and AD) of the major upper limb nerves in healthy adults. Thereafter, we planned to explore how DTI metrics varied with age, anatomical location, and experimental conditions, such as the b-value(s), echo time(s) (TE), repetition times (TR), resolution (in cubic millimetres, mm^3^) and the number of diffusion encoding gradient directions (N_D_) sampled per shell.

When the anatomical location was described we categorised data into 3 distinct regions: (1) the arm, which included data distal to the shoulder joint and ~ 5 cm proximal to the elbow joint; (2) the elbow, which included data ~ 5 cm either side of the elbow joint; and (c) the forearm, defined as 5 cm distal to the elbow joint and proximal to the radiocarpal joint.

### Methodological quality assessment

There is no consensus on the appropriate tool to assess the risk of methodological bias in observational studies of healthy volunteers, so no risk of bias assessment was performed.

### Statistical analysis

The datasets generated and/or analysed during the current study (including the outputs of metaDigitise and additional data shared by authors) are available in the Open Science Framework repository, https://osf.io/8yzst/. The PRISMA2020 tool^30^ was used to create the flow diagram. Two studies were excluded from the meta-analysis given that they were performed at 1.5T^45^ and 7T^31^. Data was analysed in Stata/MP v17 (StataCop LLC, Texas) and graphs customised using grstyle^32,33^. Using the *meta* suite, the aggregate mean FA, MD, RD and AD from studies were pooled to estimate the normative values, subgrouped by anatomical location and nerve. Restricted maximum likelihood was used to estimate the between-study variance (tau^2^), with the Knapp and Hartung modification. Heterogeneity was quantified by I^2^^34^. Sensitivity leave-one-out meta-analyses were performed to identify potential outlier studies. Mixed-effects meta-regression was then used to explore heterogeneity with FA as the dependent variable. We selected the moderator variables in the protocol phase through the production of a directed acyclic graph (http://dagitty.net/dags.html?id=cgJvh9#)^35^. The continuous fixed effects were age, resolution (mm^3^), echo time (TE in ms), b-value (s/mm^2^) and number of diffusion encoding gradient directions (N_D_) whilst the categorical fixed effect was the location (arm, elbow and forearm). For each moderator (fixed-effect), we used the minimum adjustment dataset as prescribed by DAGitty (eFigs. S1–S7). Thereafter, variance inflation factors (used to quantify potential multicollinearity) were calculated^36,37^. Confidence intervals (CI) were generated to the 95% level.

## Results

Overall, 20 studies^38^ were included (eFig. S8).

### Study characteristics

Study characteristics are detailed in eTable S1. We included data from 391 limbs belonging to 346 adults (189 males and 154 females [3 were of unknown sex], translating to ~ 1.2 M:1F) of mean age 34 years (median 31, range 20–80). The median number of authors per paper was 7 (range 5–10).

Studies were performed most commonly on Siemens (9 studies^31,38–43^, 43%) or Philips (9 studies^29,44,44–51^, 43%) scanners with the remainder using GE (3 studies^28,52,53^, 14%). Nineteen studies were performed at 3T^28,29,38–44,46–54^, with one at 1.5T^45^ and another at 7T^31^. The majority of studies used single-shot echo planar imaging (ssEPI; 17 studies, 85%), one compared ssEPI to readout-segmented echo planar imaging (rsEPI)^39^ and 3 studies^28,31,40^ did not specify the type of sequence. Subjects were most commonly in the “superman” position (i.e., prone with the shoulder and elbow extended, with the elbow positioned in the isocentre of the magnet; 17 studies^28,29,31,38,38,39,41–43,45,46,48–52,54^, 85%) whilst the others positioned individuals supine with their arm extended overhead^40^, supine in the anatomical position^44,47^ or did not report the position^53^. The receiver coils used were most commonly flexible extremity coils (11 studies^29,31,39,41–44,47,51,54^, 55%) with the remainder using head^48^, knee^38,46,50,52^, wrist^28,45^ or unspecified^53^ coils. The receiver coils had a median 8 channels (IQR 8–12, range 2–32).

The mean TE and TR were 82 ms (range 65, 105) and 5459 ms (range 2800, 10,000), respectively. The mean in-plane resolution was 1.17mm^2^ (range 0.12–1.8). The mean slice thickness was 3.5 mm (range 2–4). This generated a mean voxel volume of 5.11 mm^3^ (range 0.06–9.72). Nine studies used parallel imaging techniques (GRAPPA^39–42,42^, SENSE^29,29,49–51^, ASSET^52^, and one unspecified method^31^) whilst 9 studies^28,38,44–48,53,54^ did not specify this parameter. Four studies used partial Fourier acquisition^39–41,51^, one reported full k-space acquisition^52^ but the majority of studies^28,29,31,38,42–50,53,54^ did not report this information. Nineteen studies captured a single (maximum) b-value of mean 1045 s/mm^2^ (range 700–1300). One study^52^ captured several b-values (300, 450, 600, 750 and 900 s/mm^2^) to calculate track-weighted DTI metrics via multi-shell multi-tissue constrained spherical deconvolution. All other studies reconstructed their data using 2^nd^ order tensors. The median N_D_ was 20 (range 6–64). Most studies did not specify the diffusion encoding waveform^28,29,31,42–51,53,54^ whilst 6 studies^38–41,52^ used “monopolar” with no further explanation.

Four studies reported preprocessing their data^29,31,41,52,52^. This included MC-PCA denoising^52^; Gibbs ringing correction^52^; correction of artefacts related to susceptibility, motion and eddy currents using MRtrix3^52^, FSL^31,41^ or ExploreDTI^55^; bias correction using Advanced Normalisation Tools^52^; and interpolation of slice thickness from 3 to 1 mm^52^. Two studies^42,51^ had a single image reader/reporter whilst the remainder had two reporting clinicians/scientists^28,29,31,38,38–41,43–50,52–54^.

### Evidence synthesis: the arm

**Figure 1:**
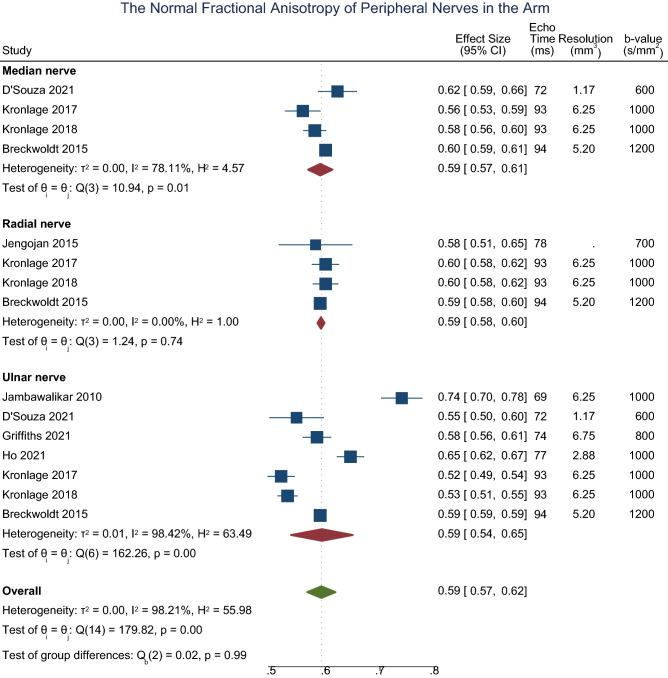
Forest plot of the normal FA of the median, ulnar and radial nerves in the arm, sorted by the echo time and b-value.

In the arm, the normal FA of the median^40,42,43,52^, radial^40,42,43,47^ and ulnar^40–42,42,46,52,54^ nerves are shown in Fig. 1. There was no significant difference in FA between the three nerves (p = 0.554, I^2^ 96%). The normal MD of the median^40,42,43,52^, radial^40,42,43,47,47^ and ulnar^40–43,52,54^ nerves was 1.13 × 10^–3^ mm^2^/s (CI 1.08, 1.18; Fig. 1) with no significant difference between nerves (p = 0.95, I^2^ 99%). The normal RD of the median^40,42,52^, radial^40,42^ and ulnar^40–42,52^ nerves was 0.70 × 10^–3^ mm^2^/s (CI 0.73, 0.76; Fig. 1) with no significant difference between nerves (p = 0.80, I^2^ 95%). The normal AD of the median^40,42,52^, radial^40,42^ and ulnar^40–42,52^ nerves was 1.99 × 10^–3^ mm^2^/s (CI 1.91, 2.08; eFigure S10) with no significant difference between nerves (p = 0.74, I^2^ 99%). Leave-one-out meta-analysis did not detect any outlier studies.

### Evidence synthesis: the elbow

**Figure 2:**
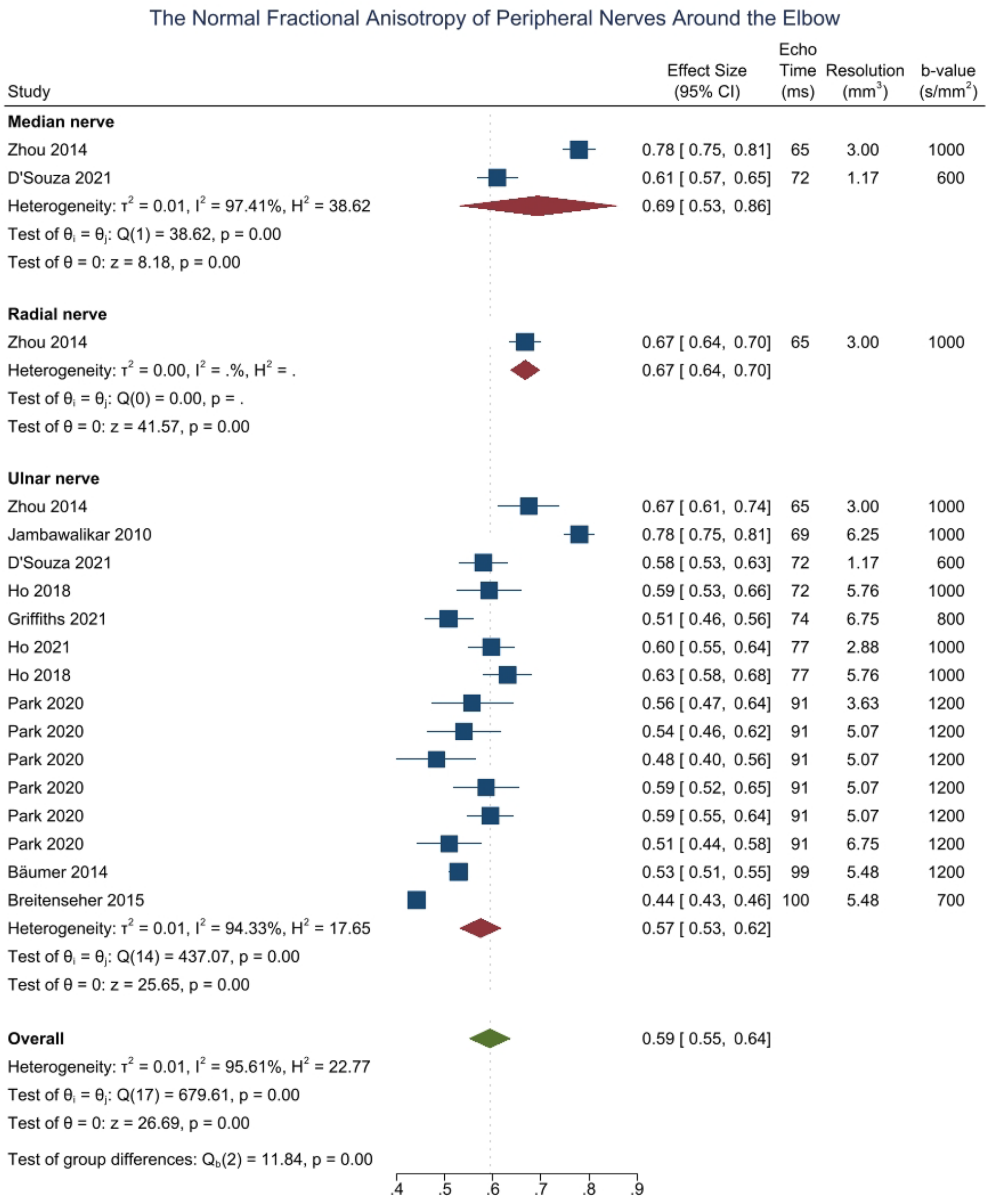
Forest plot of the normal FA of the median, ulnar and radial nerves around the elbow, sorted by the echo time and resolution.

Around the elbow, the normal FA of the median^51,52^, radial^51^ and ulnar^38,39,41,44,46,49,51,52,54^ nerves are shown in Fig. 2.

In the region of the elbow, the ulnar nerve appeared to have a 12% lower FA than the median and radial nerves (CI − 0.25, 0.00; p < 0.001, I^2^ 96%). The normal MD of the median^51,52^, radial^51^ and ulnar^39,41,44,46,49,51,52,54,54^ nerves in the elbow region was 1.01 × 10^–3^ mm^2^/s (CI 0.68, 1.34), 0.71 × 10^–3^ mm^2^/s (CI 0.64, 0.78) and 1.11 × 10^–3^ mm^2^/s (CI 1.01, 1.20), respectively (eFigure S12). The radial nerve had lower MD than the ulnar nerve (− 0.40 × 10^–3^ mm^2^/s CI − 0.76, − 0.04]; p < 0.001, I^2^ 93%) but was similar to the median nerve. The normal RD of the median^51,52^, radial^51^ and ulnar^41,51,52^ nerves around the elbow was 0.57 × 10^–3^ mm^2^/s (CI 0.41, 0.71; eFig. S13) with no significant difference between the nerves (p = 0.409). The normal AD of the median^51,52^, radial^51^ and ulnar^41,51,52^ nerves at the level of the elbow was 1.94 × 10^–3^ mm^2^/s (CI 1.71, 2.17), 1.35 × 10^–3^ mm^2^/s (CI 1.23, 1.47) and 1.88 × 10^–3^ mm^2^/s (CI 1.74, 2.03), respectively (eFig. S14). The radial nerve had a lower AD than both the ulnar nerve (mean difference 0.53 × 10^–3^ mm^2^/s [ CI 0.25, 0.82]) and median nerve (mean difference 0.59 [CI 0.28, 0.91]; p < 0.001, I^2^ 90%). Leave-one-out meta-analysis did not detect any outlier studies.

### Evidence synthesis: the forearm

**Figure 3:**
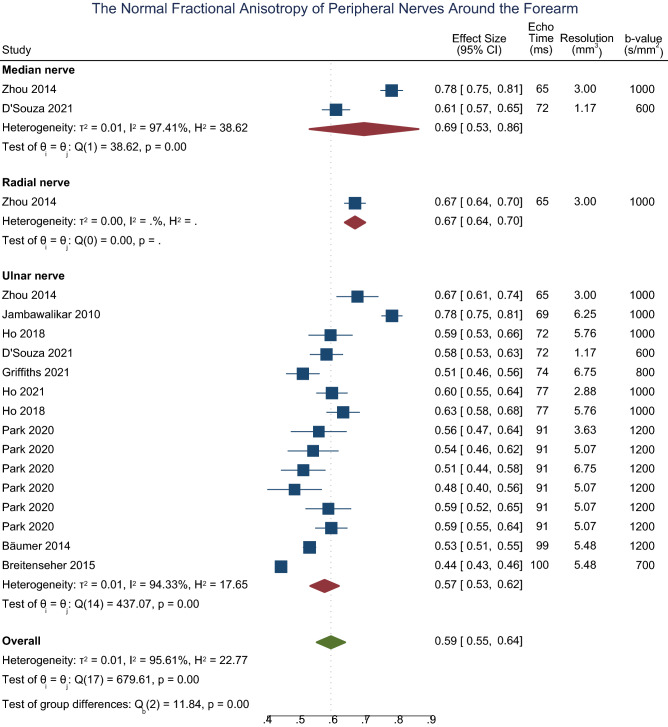
Forest plot of the normal FA of the median, ulnar and radial nerves within the forearm, sorted by the echo time and b-value.

In the forearm, the normal FA of the median^51,52^, radial^51^ and ulnar^38,39,39,41,44,46,49,51,52,54^ nerves are shown in Fig. 3 and there was no significant difference between the nerves (p = 0.690, I^2^ 96%). The normal MD of the median^29,48,50,51,53^, radial^51^ and ulnar^29,41,46,50,51^ nerves around the elbow was 1.03 × 10^–3^ mm^2^/s (CI 0.94, 1.12; eFig. S15) with no significant difference between the nerves (p = 0.409, I^2^ 97%). The normal RD of the median, radial and ulnar nerves in the forearm was 0.64 × 10^–3^ mm^2^/s (CI 0.51, 0.77; eFig. S16) with no significant difference between the nerves (p = 0.752, I^2^ 98%). The normal AD of the median, radial and ulnar nerves in the forearm was 1.91 × 10^–3^ mm^2^/s (CI 1.77, 2.04; eFig. S17) with no significant difference between the nerves (p = 0.562, I^2^ 95%). Leave-one-out meta-analysis did not detect any outlier studies.

### Meta-regression

**Figure 4:**
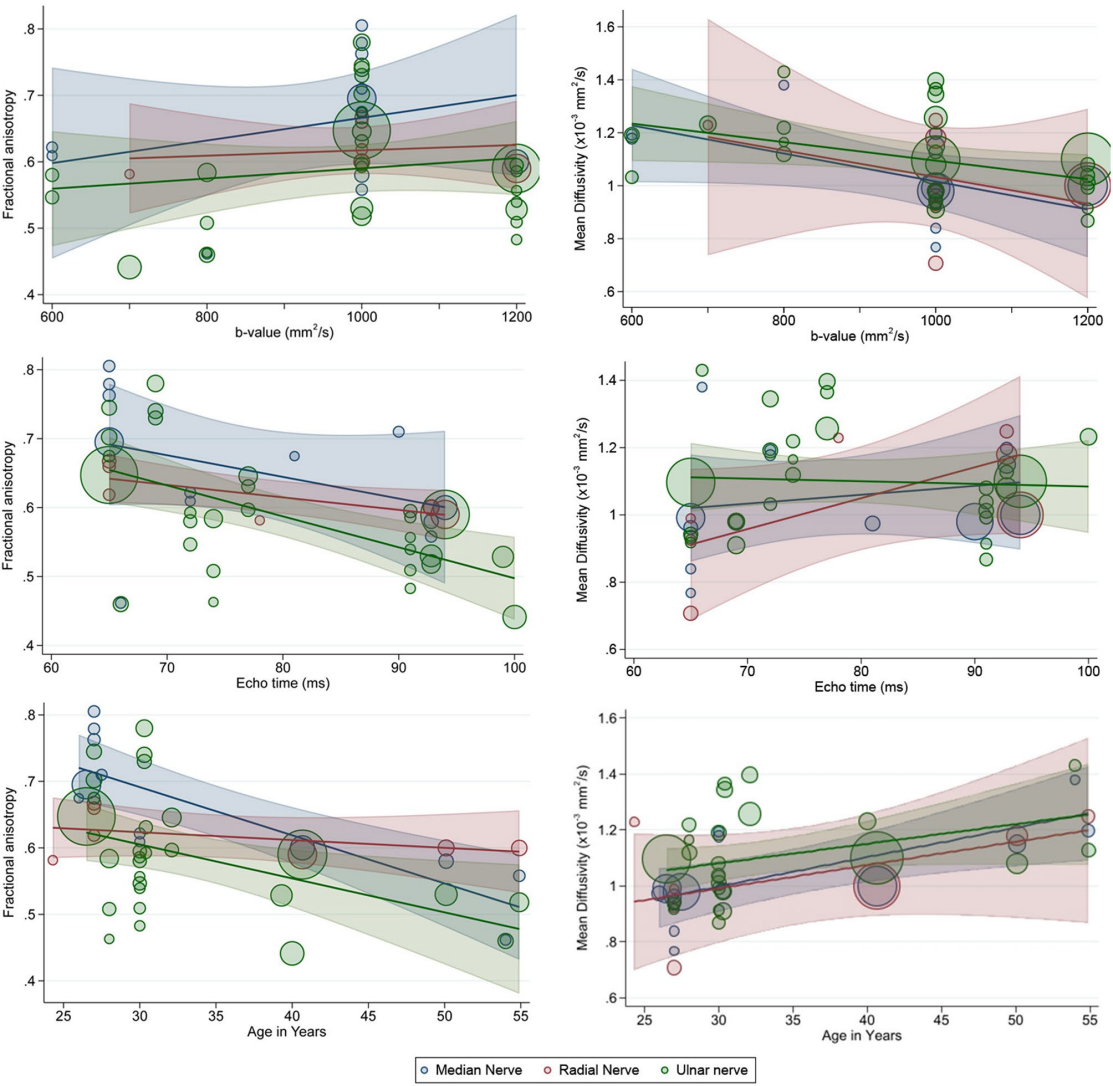
Bubble plots showing the linear dependence of FA (left column) and MD (right column) on the maximum b-value, echo time and aggregate mean age of participants. The size of the points corresponds to the precision (inverse variance) of the study.

Multivariable meta regression showed that the TE, TR, b-value, spatial resolution, anatomical location and age of the subject moderated DTI metrics within peripheral nerves (Table 1 and Fig. 4).

**Table 1:** Multivariable meta-regression of factors associated with FA and MD.

Moderators	FA	MD
	Adjusted β (95% CI)	Adjusted β (95% CI)
Echo time (ms)	− 5.6 × 10^–3^ (− 7.9 × 10^–3^, − 3.3 × 10^–3^)	5.5 × 10^–3^ (− 5.6 × 10^–4^, 0.01)
Age in years	− 4.8 × 10^–3^ (− 7.1 × 10^–3^, − 2.4 × 10^–3^)	7.8 × 10^–6^ (3.3 × 10^–6^, 1.2 × 10^–5^)
b-value (s/mm^2^)	8.9 × 10^–5^ (− 5.9 × 10^–5^, 2.4 × 10^–4^)	− 3.8 × 10^–4^ (− 6.4 × 10^–4^, − 1.2 × 10^–4^)
Location		
Arm	Referent	Referent
Elbow	1.5 × 10^–3^ (− 0.06, 0.06)	− 0.06 (− 0.17, 0.05)
Forearm	0.06 (2.5 × 10^–4^, 0.13)	− 0.10 (− 0.17, 0.05)
Repetition time (s)	7.9 × 10^–6^ (− 1.4 × 10^–5^, 3.0 × 10^–5^)	− 5.1 × 10^–5^ (− 9.3 × 10^–5^, − 9.6 × 10^–6^)
Resolution (mm^3^)	− 0.02 (− 0.03, − 0.004)	0.02 (9.1 × 10^–4^, 0.04)
N_D_	1.8 × 10^–3^ (− 3.9 × 10^–4^, 4.0 × 10^–3^)	3.6 × 10^–3^ (− 7.8 × 10^–3^, 4.8 × 10^–4^)

N_D_ = the number of diffusion encoding gradient directions.

Note that each variable has been differently adjusted, as defined by our DAGs (http://dagitty.net/mcgJvh9, eFigs. S11–S17).

Both anisotropy and diffusivity were dependent on age whereby each decade of life reduced the FA by 0.05 (CI 0.007, 0.02) and increased MD by 7.8 × 10^–5^ mm^2^/s (CI 3.3 × 10^–5^, 1.2 × 10^–4^). Increments in the b-value of 100 s/mm^2^ reduced the observed MD by approximately 0.038 mm^2^/s (CI 0.064–0.012) without affecting the FA. increments of 10 ms in the TE reduced the FA within peripheral nerves by approximately 0.056 (CI 0.079, 0.033) without affecting the MD. Increasing the spatial resolution by 1mm^3^ downwardly biased the FA by 2% (CI 3–4) and upwardly biased the MD by 0.02 (CI 9.1 × 10^–4^, 0.04). The nerves within the forearm had a 6% higher FA than nerves within the arm. FA and MD appeared to be robust to N_D_.

## Discussion

This work shows that dMRI metrics from healthy nerves in the upper limb are dependent on experimental conditions and age, and differ throughout the length of the limb. Importantly, we show that seemingly small alterations to acquisition parameters (e.g., changing the b-value by 100 mm^2^/s or TE by 10 ms) is associated with meaningful changes to DTI measurements. Equally, we show that DTI metrics from the median, ulnar and radial nerves are age-dependent, which has important ramifications.

Our work corroborates prior dMRI studies in peripheral nerves^16,17^ (and the brain^56^) which demonstrate that nerves exhibit more isotropic diffusion with advancing age. This is expected because aging axons lose their integrity, axoplasmic transport is slowed and the myelin sheath deteriorates which gives way to segmental demyelination and axonal loss without remyeliation^57^. These morphological changes lead to an increase in extra-cellular fluid and decline in both the density and integrity of microstructures which hinder/restrict diffusion. On a practical level, the observation that aging nerves exhibit more isotropic diffusion is important because it shows that when comparing group differences or longitudinal changes in dMRI metrics, adjustment for age is likely to be required.

In keeping with the prior literature, this work solidifies a clinically important and unique features of the pattern of diffusion within the ulnar nerve. At the level of the elbow and more distally within the forearm, diffusion within the ulnar nerve appears to become more isotropic compared to its proximal course. This may be a manifestation of microstructural changes due to hardships endured around the elbow, namely repetitive mechanical deformation as the elbow moves, cumulative external trauma from knocks (aka the “funny bone”) and the resistance created due to the passage through a relatively tight fibrosseous (cubital) tunnel. These factors together may contribute to systematic differences in the microstructure of the ulnar nerve which render its diffusion more isotropic within and beyond the cubital tunnel.

In our multivariable meta-regression model, prolongation of the TE was associated with lower estimates of anisotropy. Whilst we show the same lack of association between TE and MD as observed in the brain^58^, the observation that TE downwardly biases FA in the limb does not agree with the literature in the human brain (at both 1.5 and 3 T^58^) whereby a positive linear correlation was observed between TE and the FA within white matter. However, the opposite was observed in this study, which is difficult to explain, so we offer some hypotheses. We observed a positive linear relationship between TE and aggregate age (β 0.40 [CI 0.21, 0.58]) which might explain why studies with longer TE (older participants) had lower FA. Secondly, there was a linear correlation between TE and b-value (r = 0.42) but the variance inflation factor for TE and b-value was 4.9 and 1.2, respectively in the model with TE as the exposure. Consensus amongst the statistical community is that a correlation coefficient > 0.7 between predictor variables or variance inflation factors > 10 is evidence of multicollinearity and should lead to the exclusion of colinear variables. Our models appear to have some collinearity (not enough to warrant variable removal) and we feel that this might contribute but does not completely explain the relationship between TE and FA. Finally, FA decreasing with TE might represent differences in the T_2_ of intracellular water and extracellular water in peripheral nerves. Compounding this is the problem of myelin’s magnetic susceptibility which alters the off-resonance field^59^ for intra-axonal water, meaning that at longer echo times (after diffusion-weighting) there may be phase offsets which amplify differences between intra- and extra-axonal water. Also, at longer echo times there may be more sensitivity to non-gaussian diffusion in peripheral nerves, which has been observed to start from lower b-values than in the brain (~ 700 s/mm^2^)^60^. Finally, the included studies did not report the diffusion time (and many other important methods), so it is plausible that the relationship between TE and FA was confounded by something else. Future studies should seek to: (a) fully report the parameters of their sequences, (b) report methods of pre- and postprocessing, and (c) make their anonymised data available open source to enable individual patient-data meta-analysis.

There are some important limitations to our study. Non-gaussian diffusion has been observed at b-values above ~700 s/mm^2^ and consequently, monoexponential fitting (i.e., a 2nd order tensor) may be influenced by restricted diffusion at higher b-values^60^. It is widely accepted that preprocessing of dMRI data improves the accuracy of metrics and tractography^61^, and differences in preprocessing practices and pipelines generate important differences in results^2^ which negatively impacts reproducibility^15^. In this review, most studies failed to report if or what preprocessing was performed, and this may be a source of variability. Finally, few authors described their postprocessing methods (e.g. the size and position of regions-of-interest used to extract DTI metrics, how they were drawn, etc.) which is important because recent work has shown that subtle variability in the size and position of regions of interest have downstream effects on DTI metrics^10^. Some readers may decry our decision to meta-analyse statistically heterogeneous data, but this was done purposively because forest plots provide an important graphical representation of measurement variation in relation to experimental conditions (e.g., b-values and N_D_) and they summarise a large amount of information in an easy-to-interpret format. Furthermore, by making this choice we could deploy meta-regression to explore potential moderators. Ultimately, our choice to meta-analyse heterogenous data has provided important insight into factors which appear to moderate FA and diffusivity within the nerves of the upper limb.

Non-biological variability in dMRI metrics undermines the reliability of multi-site and/or longitudinal studies. Therefore, there remains a need for robust harmonisation techniques^62,63^. Harmonisation is a mathematical approach (regression, interpolation or machine learning) which seeks to reduce the unwanted (non-biological) variability in dMRI datasets whilst retaining information which pertains to the underlying microstructure and physiology^64^. A recent review of Harmonisation of dMRI^64^ showed the benefits of such an approach. By summarising the effects of non-biological variability in dMRI of the arm, elbow and forearm, we provide information which may inform harmonisation efforts in the limb by quantifying the direction and magnitude of dMRI metric variation in relation to non-biological factors.

In conclusion, we show that dMRI metrics from healthy nerves in the upper limb are age-dependent, and that the b-value, echo time, repetition time and resolution are clinically important sources of variability. We provide summary estimates of the normal values of the median, ulnar and radial nerves in different experimental settings which may be of value to researchers and clinicians alike.

## Supplementary Information

Supplementary Information.

## Data Availability

The datasets generated and/or analysed during the current study are available in the Open Science Framework repository, https://osf.io/8yzst/.
