# Supplementary material for: Meta-analysis of the normal diffusion tensor imaging values of the peripheral nerves in the upper limb
Source: Sci Rep. 2023 Mar 24;13:4852. doi: 10.1038/s41598-023-31307-2 (PMC10039047; doi:10.1038/s41598-023-31307-2)
Supplement: Supplementary file 1 — Supplementary Information. [file 41598_2023_31307_MOESM1_ESM.docx]

**Supplementary Materials**

eTable 1. Study characteristics. ¥ Information not specified. SS = single shot. RS = read-out-segment

| **Study** | **Country** | **Make &**  **Model (T)** | **TE (TR) in ms** | **EPI type** | **Subject position (coil [Transmit/Receive T/R or Receive only, R], channels)** | **Parallel Imaging (partial Fourier)** | **Resolution (In plane x Slice Thickness, mm)** | **Number of Signals** | **b-values (directions)** | **Number of raters** | **Subjects (limbs) M:F** | **Mean Age (SD) [Range]** |
| --- | --- | --- | --- | --- | --- | --- | --- | --- | --- | --- | --- | --- |
| Bäumer 2014^37^ | Germany | Siemens  TIM-TRIO (3T) | 99 (3800) | SS | Superman (Knee [T/R], 15) | ¥ (¥) | 1.17x1.17x4 | 2 | 1200 (19) | 2 | 30 (30) 13:17 | 39.3 (11.38) [25-68] |
| Ohana 2012^27^ | France | GE  Signa (3T) | 105 (5200) | ¥ | Superman (Wrist [R], 8) | ¥ (¥) | 1.09x1.09x4 | 3 | 1000 (15, 20, 30) | 2 | 14 (14) 11:3 | 27.5 (1.76) [20-32] |
| Jengojan 2015^46^ | Austria | Philips  Achieva (3T) | 78 (6433) | SS | Supine, arm by side (Flexible [R], ¥) | ¥ (¥) | 1.5x1.5x ¥ | 5 | 700 (16) | 2 | 13 (13) 8:5 | 24.3(1.94) [22-28] |
| Ho 2021^53^ | Switzerland | Siemens  Skyra (3T) | 77 (4400) | SS | Superman (Flexible, 4) | ¥ (¥) | 1.2x1.2x2 | 1 | 1000 (20) | 2 | 10 (20) 5:5 | 32.1 (4.45) [¥-¥] |
| Ho 2018^38^ | Switzerland | Siemens  Skyra (3T) | 77 (4400) | SS and RS | Superman (Flexible, 4) | GRAPPA (6/8) | 1.2x1.2x4 | 2 | 1000 (20) | 2 | 10 (10) 5:5 | 30.4 (4.01) [25-36] |
| Park 2020^48^ | Korea | Philips  Achieva (3T) | 91 (7049) | SS | Superman (¥, ¥) | SENSE (¥) | 1.1x1.1x3  1.3x1.3x3  1.5x1.5x3  1.3x1.3x4  1.3x1.3x5 | 5 | 1200 (15) | 2 | 15 (15) 9:6 | 30 (6.8) [22-42] |
| Breckwoldt 2015^39^ | Germany | Siemens  TIM-TRIO and Vario (3T) | 94 (3800) | ¥ | Supine arm overhead (¥, ¥) | GRAPPA (7/8) | 1.14x1.14x4 | 2 | 1200 (19) | 2 | 30 (30) 16:14 | 40.67 (13.07) [20-70] |
| Kabakci 2007^47^ | Turkey | Philips  Achieva (3T) | 90 (4600) | SS | Superman (head [T/R], 8] | ¥ (¥) | 1.09375x1.09375x4 | 3 | 1000 (32) | ¥ | 20 (20) 6:14 | 27.5 (1.85) [21-35] |
| Kronlage 2017^41^ | Germany | Siemens  TIM-TRIO (3T) | 92.8 (4000) | SS | Superman (Flexible [R], 16) | GRAPPA (¥) | 1.25x1.25x4 | 3 | 1000 (20) | 1 | 18 (18) 11:7 | 54.9 (13.3) [¥-¥] |
| Zhou 2012^49^ | USA | Philips  Achieva (3T) | 65 (7200) | SS | Superman (Knee [T/R], 8) | SENSE (¥) | 1.8x1.8x3 | 2 | 1000 (21) | ¥ | 10 (20) 5:5 | 26.5 (3.23) [22-32] |
| Jambawalikar 2010^45^ | USA | Philips  ¥ (3T) | 69 (5300) | SS | Superman (Knee [T/R], 8) | ¥ (¥) | 1.25x1.25x4 | 2 | 1000 (6) | ¥ | 10 (10) 8:2 | 30.3 (4.85) [24-39] |
| Stein 2009^52^ | Israel | GE Signa | 81 (10,000) | SS | ¥ (¥,¥) | ¥ (¥) | ¥ x1.09x3 | 2 | 1000 (6) | ¥ | 17 (17) 7:10 | 26 (2.78) [24-34] |
| Zhou 2014^50^ | USA | Philips  ¥ (3T) | 65 (7200) | SS | Superman (Flexible [R], 8) | SENSE (6/8) | 1x1x3 | 2 | 1000 (42) | 1 | 10 (10) 5:5 | 27 (3.88) [22-34] |
| Breitenseher 2015^43^ | Austria | Philips  Achieva (3T) | 100 (3964) | SS | Supine, arm by side (Flexible [R], 2) | ¥ (¥) | 1.18x1.16x4 | 2 | 700 (16) | 2 | 20 (20) 14:6 | 40 (8.56) [28-60] |
| Haakma 2017^28^ | Netherlands | Philips  Achieva (3T) | 66 (6340) | SS | Superman (Flexible [R], 32) | SENSE (¥) | 1.5x1.5x4 | 1 | 800 (15) | ¥ | 10 (20) 8:2 | 54 (12.28) [29-67] |
| Kronlage 2018^42^ | Germany | Siemens  TIM-TRIO (3T) | 92.8 (4000) | SS | Superman (Flexible [R], 16) | GRAPPA (¥) | 1.25x1.25x4 | 3 | 1000 (20) | 2 | 60 (60) 30:30 | 50.1 (17. 3) [25-80] |
| Raval 2017^30^ | USA | Siemens  TIM-TRIO (7T) | 83 (7000) | ¥ | Superman (Flexible [R], 8) | Used but ¥ (¥) | 0.14x0.14x3 | 5 | 1300 (64) | 2 | 3 (3) ¥:¥ | ¥ (¥) [¥-¥] |
| Griffiths 2021^40^ | UK | Siemens  Prisma (3T) | 74 (7800) | SS | Superman (Flexible [R], 4) | GRAPPA (6/8) | 1.5x1.5x3 | 4 | 800 (30) | 2 | 13 (13) 8:5 | 28 (6) [21-40] |
| D'Souza 2021^51^ | Australia | GE  MR750 (3T) | 72 (3900) | SS | Superman (Knee [T/R], 8) | ASSET (Not used) | 0.625x0.625x3 | 2 | 300, 450, 600, 750 and 900 (24) | ¥ | 8 (8) 5:3 | 30 (8) [¥-¥] |
| Edward 2020^44^ | Egypt | Philips  Sense (1.5T) | ¥ (¥) | SS | Superman (Wrist, ¥) | ¥ (¥) | ¥ | ¥ | 2000 (32) | ¥ | 15 (30) 10:5 | 45.67 (7.77) [¥-¥] |

**eFigure 1.** The directed acyclic graph designed to define confounders (red coloured nodes, with red arrows) which must be adjusted for when exploring the relationship between b-value (the exposure) and fractional anisotropy (the outcome). Open causal pathways have green arrows. TE = echo time, TR = repetition time, RoI = region of interest, FA = fractional anisotropy.


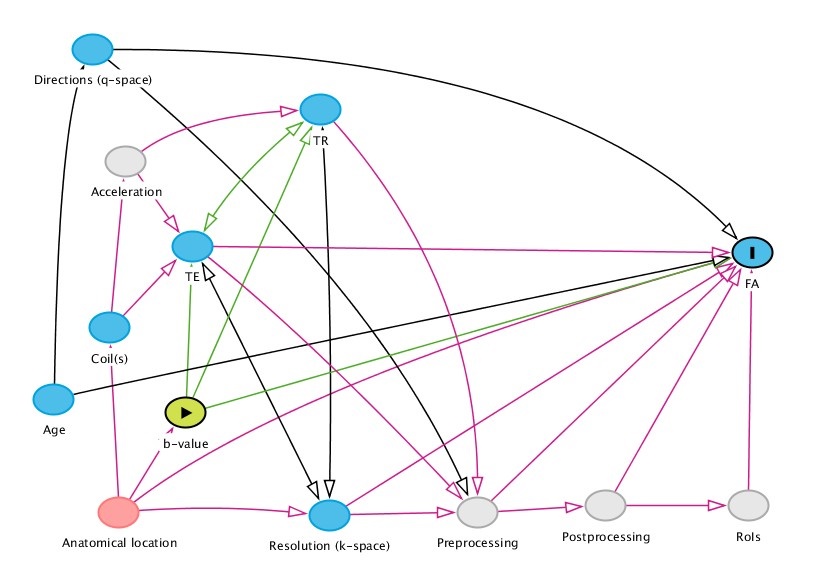


eFigure 2. The directed acyclic graph designed to define confounders (red coloured nodes, with red arrows) which must be adjusted for when exploring the relationship between TE (the exposure) and fractional anisotropy (the outcome). Open causal pathways have green arrows. TE = echo time, TR = repetition time, RoI = region of interest, FA = fractional anisotropy.


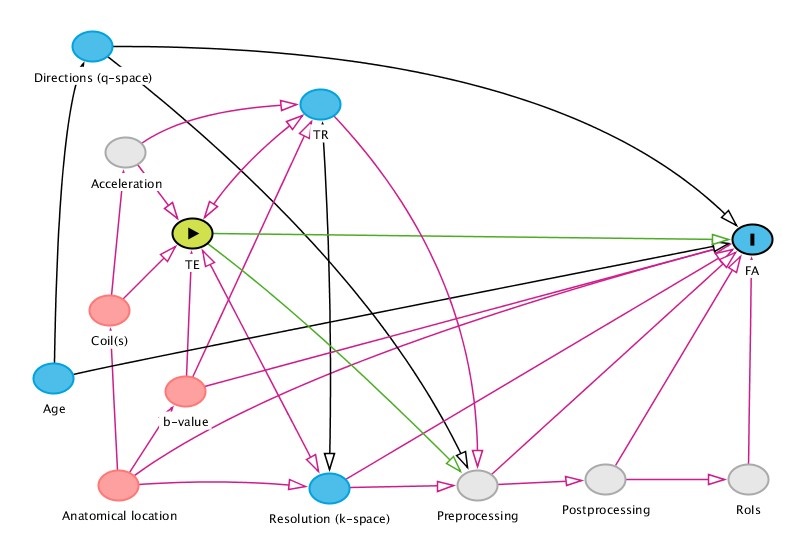


eFigure 3. The directed acyclic graph designed to define confounders (of which there are none in this network) which must be adjusted for when exploring the relationship between age (the exposure) and fractional anisotropy (the outcome). Open causal pathways have green arrows. TE = echo time, TR = repetition time, RoI = region of interest, FA = fractional anisotropy.


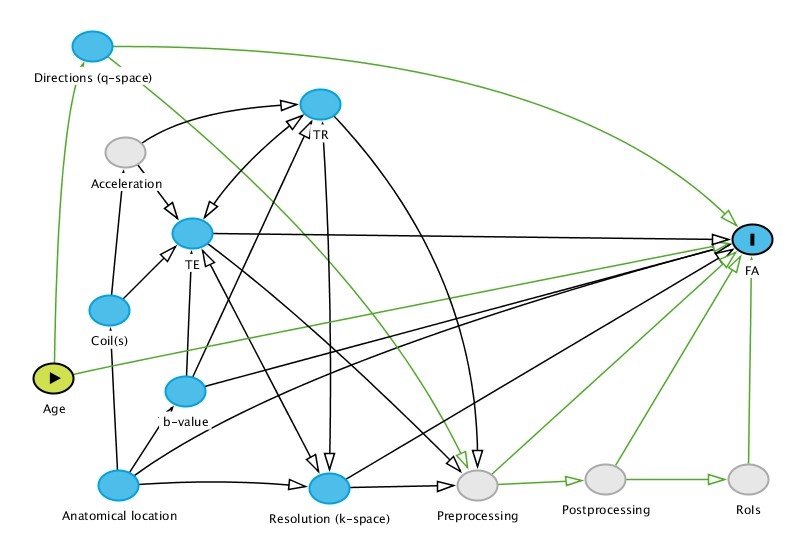


eFigure 4. The directed acyclic graph designed to define confounders (of which there are none) which must be adjusted for when exploring the relationship between anatomical location (the exposure) and fractional anisotropy (the outcome). Open causal pathways have green arrows. TE = echo time, TR = repetition time, RoI = region of interest, FA = fractional anisotropy.


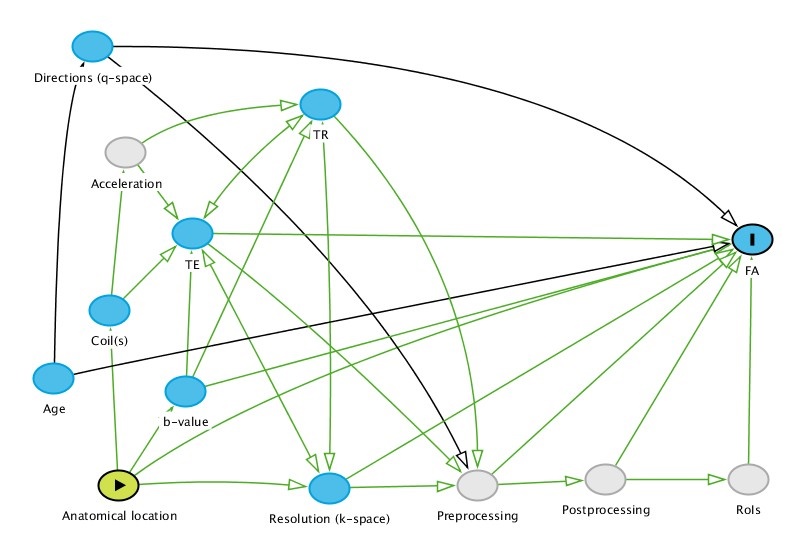


eFigure 5. The directed acyclic graph designed to define confounders (red coloured nodes, with red arrows) which must be adjusted for when exploring the relationship between TR (the exposure) and fractional anisotropy (the outcome). Open causal pathways have green arrows. TE = echo time, TR = repetition time, RoI = region of interest, FA = fractional anisotropy.


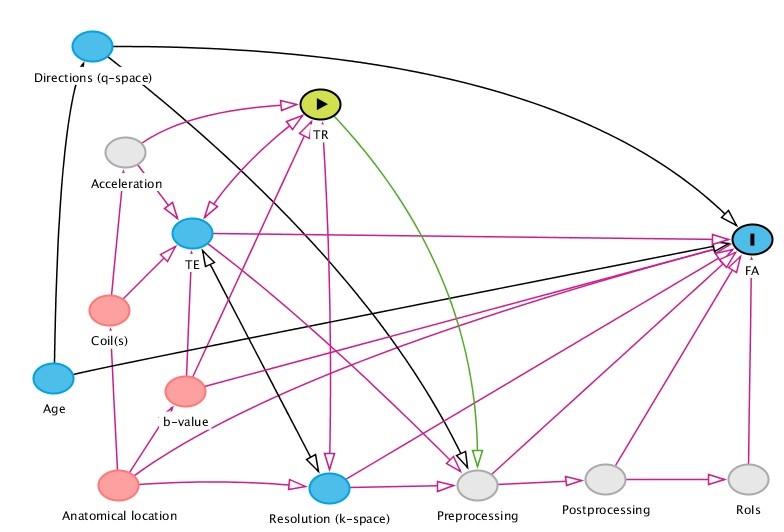


eFigure 6. The directed acyclic graph designed to define confounders (red coloured nodes, with red arrows) which must be adjusted for when exploring the relationship between spatial resolution (the exposure) and fractional anisotropy (the outcome). Open causal pathways have green arrows. TE = echo time, TR = repetition time, RoI = region of interest, FA = fractional anisotropy.


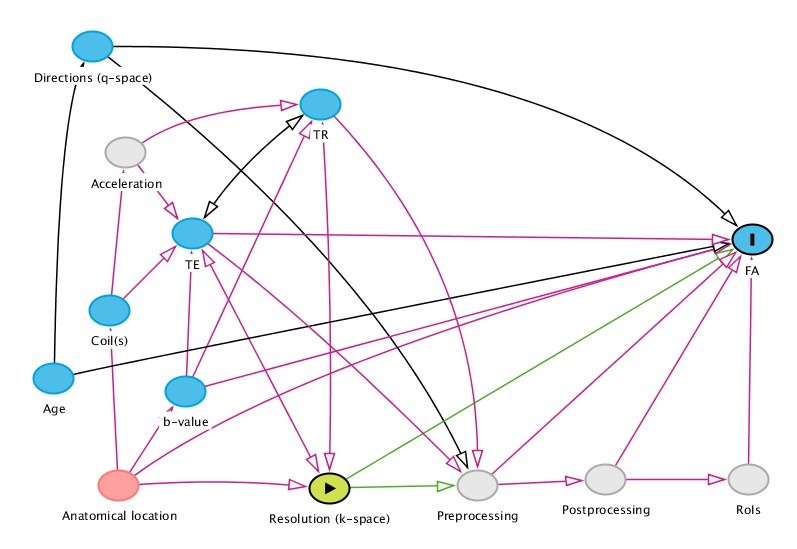


eFigure 7. The directed acyclic graph designed to define confounders (red coloured nodes, with red arrows) which must be adjusted for when exploring the relationship between N_D_ (the exposure) and fractional anisotropy (the outcome). Open causal pathways have green arrows. TE = echo time, TR = repetition time, RoI = region of interest, FA = fractional anisotropy.


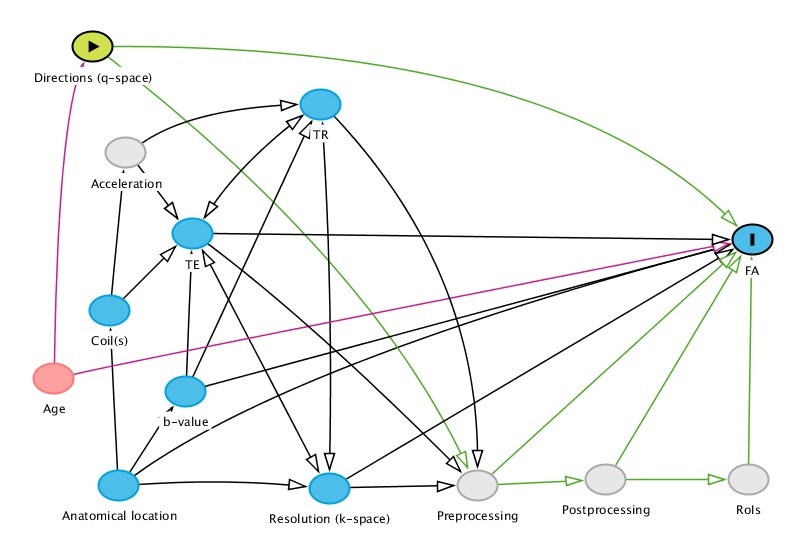


eFigure 8. PRISMA flow diagram


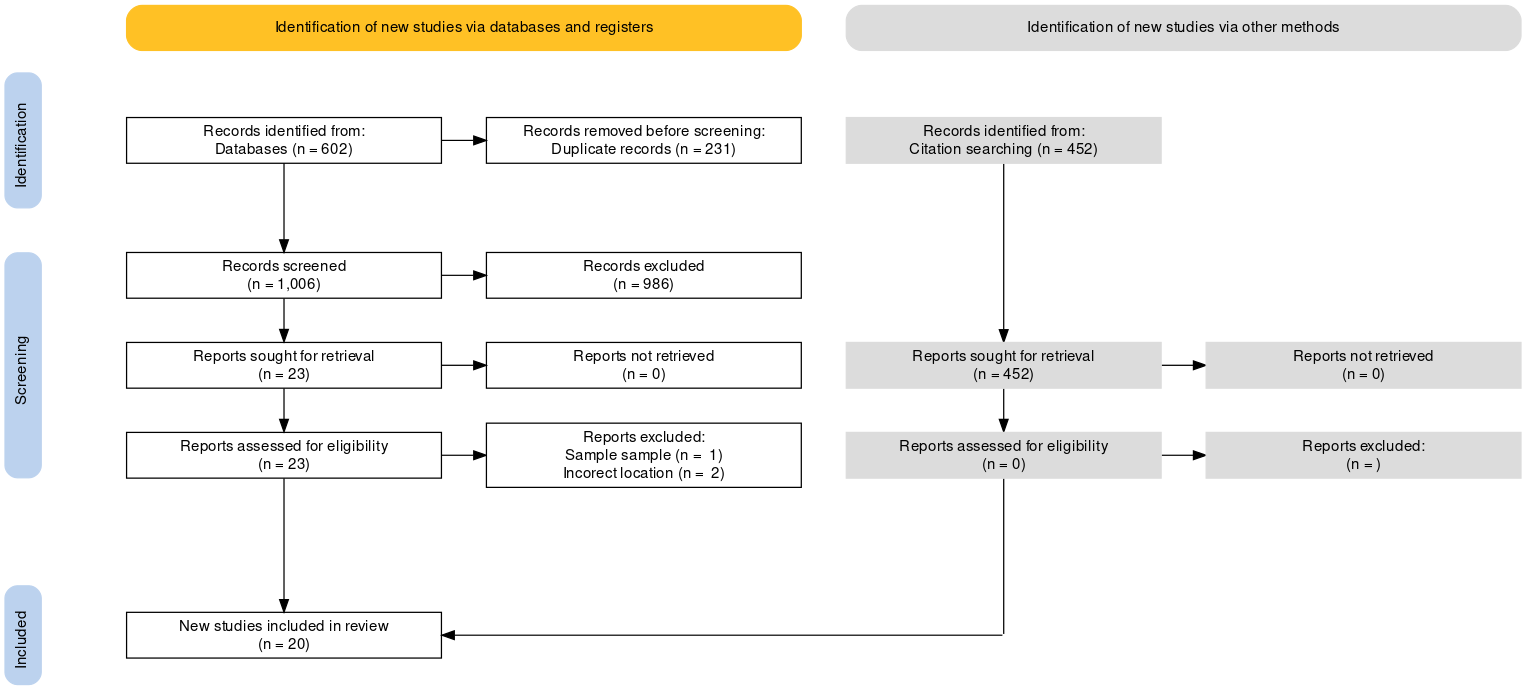


**eFigure 9.** Forest plot of the normal MD of the median, ulnar and radial nerves in the arm, sorted by the echo time and b-value.

**eFigure 10.** Forest plot of the normal RD of the median, ulnar and radial nerves in the arm, sorted by the echo time and b-value.

**eFigure 11.** Forest plot of the normal AD of the median, ulnar and radial nerves in the arm, sorted by the echo time and b-value.

**eFigure 12.** Forest plot of the normal MD of the median, ulnar and radial nerves around the elbow, sorted by the echo time and b-value.

**eFigure 13.** Forest plot of the normal RD of the median, ulnar and radial nerves around the elbow, sorted by the echo time and b-value.

**eFigure 14.** Forest plot of the normal AD of the median, ulnar and radial nerves around the elbow, sorted by the echo time and b-value.

**eFigure 15.** Forest plot of the normal MD of the median, ulnar and radial nerves in the forearm, sorted by the echo time and b-value.

**eFigure 16.** Forest plot of the normal RD of the median, ulnar and radial nerves in the forearm, sorted by the echo time and b-value.

**eFigure 17.** Forest plot of the normal AD of the median, ulnar and radial nerves in the forearm, sorted by the echo time and b-value.

**Appendix 1.** Search strategy

1. Median
2. Pronator
3. Ulnar
4. Cubital
5. Radial
6. Forearm
7. Arm
8. Upper limb
9. Elbow
10. Diffusion?tensor
11. DTI
12. Diffusion MRI
13. Diffusion magnetic resonance imaging
14. Q?space
15. Diffusion?weighted
16. DWI
17. Anisotropic diffusion
18. 1 OR 2 OR 3 OR 4 OR 5 OR 6 OR 7 OR 8 OR 9
19. 10 OR 11 OR 12 OR 13 OR 14 OR 15 OR 16 OR 17
20. 18 AND 19
